# Domestic animals as sentinels for environmental carcinogenic agents

**DOI:** 10.1186/1753-6561-7-S2-K13

**Published:** 2013-04-04

**Authors:** Katia Cristina Kimura, Danielle de Almeida Zanini, Adriana Tomoko Nishiya, Maria Lucia Zaidan Dagli

**Affiliations:** 1 School of Veterinary Medicine and Animal Science of the University of São Paulo, USP, São Paulo, Brazil; 2 Anhembi-Morumbi University, São Paulo, SP, Brazil

## 

The idea of using animals as sentinels of environmental contaminations is not new, but until now is relatively little explored. The aim of this talk is to present evidences that lymphomas in dogs of the city of São Paulo may be associated to exposure to air pollution caused by vehicle emissions and heavy traffic, and that the spatial distribution of canine lymphomas in the city of São Paulo coincides with the spatial distribution of human lymphomas in the same city.

Lymphomas are highly prevalent malignant neoplasms that affect canines, humans and other species. Several reports state that, besides a genetic breed susceptibility, environmental factors may be associated to the development of these neoplasms. One first study aimed to investigate the possible factors associated with the development of canine lymphomas. The owners of 83 dogs with lymphomas and 84 controls were interviewed through an epidemiological questionnaire. Cases and control animals lived in the same geographical areas and were admitted to the same veterinary hospitals (five locations in total), in the city of São Paulo. The results showed that age, gender, breed, diet, reproductive status, mineral or tap water, exposures as passive smoking, herbicides, insecticides, fungicide, building materials, products for home maintenance and other factors were unrelated to the risk of malignant lymphoma. Interestingly, our study detected that dogs which were permanently kept outdoors and around 100 meters to busy streets or avenues (more than 50 vehicles per minute) had a higher risk for development of the disease. The city of São Paulo, Brazil, is the largest city in South America and the fourth largest city in the world housing a fleet of about five million cars and a million motorcycles. These results suggest that environmental factors, such as the air pollution caused by the heavy traffic, may be associated to the development of canine lymphomas.

A second study has been performed in order to verify and compare the spatial distribution of canine and human lymphomas in the city of São Paulo. Non-Hodgkin`s lymphomas (NHLs) are complex and heterogeneous neoplasms characterized by malignant proliferation of lymphoid cells. Human and canine NHLs share several features. Because dogs share most environmental and living conditions with humans, we reasoned that spatial distributions of NHL cases between the two species would provide some evidence for the role of certain environmental factors, such as pollutants, in the pathogenesis of NHLs. In this study, we retrospectively analyzed the spatial distributions of 630 human NHL cases randomly selected from a database of more than 8.000 cases at the Cancer Registry of São Paulo and 579 canine NHL cases diagnosed in five referral veterinary hospitals in the city. All human and canine cases were recorded between 1996 and 2006. Here we show that human and canine cases of NHL have similar spatial distributions in the city of São Paulo, with a high incidence in the central region, which is the most polluted area of the city, due to the presence of avenues with a heavy traffic of vehicles. These results suggest that environmental pollutants may play a role in the pathogenesis of human and canine NHLs.

In conclusion, the results of these two studies have suggested that canine lymphomas, and possibly human lymphomas, may be associated with the exposure to air pollution. Other studies are being conducted to better establish the relationship between air pollution and development of human and canine lymphomas. Fortunately, the government of the state of São Paulo is aware of the deleterious effects of air pollution on human health and is taking measures to better control the levels of air pollution in the city.

## Competing interests

There are no competing interests in this presentation.

